# *Momordica charantia *(bitter melon) inhibits primary human adipocyte differentiation by modulating adipogenic genes

**DOI:** 10.1186/1472-6882-10-34

**Published:** 2010-06-29

**Authors:** Pratibha V Nerurkar, Yun-Kung Lee, Vivek R Nerurkar

**Affiliations:** 1 Laboratory of Metabolic Disorders and Alternative Medicine, Department of Molecular Biosciences and Bioengineering, College of Tropical Agriculture and Human Resources, University of Hawaii at Manoa, Honolulu, Hawaii 96822 USA; 2 Retrovirology Research Laboratory, Department of Tropical Medicine, Medical Microbiology and Pharmacology, John A. Burns School of Medicine, University of Hawaii at Manoa, Honolulu, Hawaii 96813 USA

## Abstract

**Background:**

Escalating trends of obesity and associated type 2 diabetes (T2D) has prompted an increase in the use of alternative and complementary functional foods. *Momordica charantia *or bitter melon (BM) that is traditionally used to treat diabetes and complications has been demonstrated to alleviate hyperglycemia as well as reduce adiposity in rodents. However, its effects on human adipocytes remain unknown. The objective of our study was to investigate the effects of BM juice (BMJ) on lipid accumulation and adipocyte differentiation transcription factors in primary human differentiating preadipocytes and adipocytes.

**Methods:**

Commercially available cryopreserved primary human preadipocytes were treated with and without BMJ during and after differentiation. Cytotoxicity, lipid accumulation, and adipogenic genes mRNA expression was measured by commercial enzymatic assay kits and semi-quantitative RT-PCR (RT-PCR).

**Results:**

Preadipocytes treated with varying concentrations of BMJ during differentiation demonstrated significant reduction in lipid content with a concomitant reduction in mRNA expression of adipocyte transcription factors such as, peroxisome proliferator-associated receptor γ (PPARγ) and sterol regulatory element-binding protein 1c (SREBP-1c) and adipocytokine, resistin. Similarly, adipocytes treated with BMJ for 48 h demonstrated reduced lipid content, perilipin mRNA expression, and increased lipolysis as measured by the release of glycerol.

**Conclusion:**

Our data suggests that BMJ is a potent inhibitor of lipogenesis and stimulator of lipolysis activity in human adipocytes. BMJ may therefore prove to be an effective complementary or alternative therapy to reduce adipogenesis in humans.

## Background

In the United States, approximately 127 million adults are overweight (body mass index, BMI > 25 kg/m^2^) while more than 60 million are classified as obese (BMI > 30 kg/m^2^), and about 9 million as severely obese (BMI > 40 kg/m^2^). In the last two decades, the prevalence of overweight individuals has increased by 40% (from 46.0% to 64.5%) and the prevalence of obesity has risen by 110% (from 14.5% to 30.5%) [1]. Obesity is also associated with metabolic disorders such as type 2 diabetes (T2D), which is the second leading cause of preventable death in the United States [2].

Adipose tissue is a critical endocrine organ that is innately involved in regulating not only obesity, but also metabolic processes such as, insulin resistance and type-2 diabetes mellitus (T2DM) [3,4]. Adipogenesis involves development of preadipocytes to mature adipocytes with the accumulation of lipid droplets, increase in fat cell size (hypertrophy), as well as increase in cell number or hyperplasia and plays an important role in obesity. Among the various transcription factors that promote preadipocyte differentiation and influence adipogenesis, peroxisome proliferator-activated receptor γ (PPARγ) is considered the "master regulator of adipogenesis" [5,6]. Other adipogenic transcription factors include the CCAAT/enhancer binding proteins (C/EBPα, C/EBPβ and C/EBPδ) and sterol regulatory element-binding protein 1c (SREBP-1c) [7-9]. Similarly, lipolysis or lipid mobilization in adipocytes results from breakdown of adipose triacylglycerols (TAG) into nonesterified fatty acids (NEAF) and glycerol and involves not only lipases and TAG hydrolases, but also lipid-droplet coating proteins such as perilipin [10,11]. Besides adipocyte transcription factors, adipocyte-secreted factors (adipocytokines) also play an important role in primary human preadipocyte differentiation [12]. Adipocytokine resistin, a 12-kDa peptide, is increased along with PPARγ during the differentiation of 3T3-L1 adipocytes [13] and primary human adipocytes [12]. Overall, an appropriate balance between adipogenesis and lipolysis is therefore crucial for the proper functioning of adipose tissue, which plays an important role in obesity and associated metabolic functions.

Successful treatment of obesity usually requires multiple interventions such as exercise programs, diet, behavioral modification and pharmacotherapy [14-20]. Escalating trends of obesity has resulted in a renewed interest in the use of functional foods including herbs and alternative medicine [21]. *Momordica charantia*, commonly known as bitter melon (BM), is widely cultivated in Asia, East-Africa and South America and extensively used in folk medicines as a remedy for diabetes and its complications, specifically in India, Southeast Asia, Africa and South America [22]. Animal studies indicate that BM juice (BMJ) was also effective in reducing weight gain, possibly due to reduced adipose hypertrophy, inhibition of lipogenic genes and increased plasma catecholamines [23-26]. However, effects of BMJ on human adipocyte differentiation are unknown. Therefore, the goal of our project was to elucidate the effects of BMJ on lipid accumulation, lipid mobilization, and expression of adipocyte transcription factors and adipocytokine, in primary human adipocytes. It was observed that BMJ significantly reduced lipid accumulation and increased lipolysis in primary human adipocytes, with a concomitant reduction in PPARγ, SREBP-1c, perilipin, and resistin genes expression. These studies are critical as they lay the foundation to identify molecular targets and anti-obesity effects of bitter melon for future clinical trials.

## Methods

### Preparation of Bitter Melon Juice (BMJ)

Chinese variety of young BM (which had not ripened and turned yellow/orange) was obtained from local farmer's market, washed and deseeded. BMJ was extracted according to the previously published protocol [26,27]. In brief, young, green BM fruits were deseeded and pulp was discarded. Juice was prepared in a regular household juicer and centrifuged at 4,500 rpm at 4°C for 30 min. About 8,000 g of deseeded BM fruit yielded 3,500 mL of BMJ. BMJ supernatant was aliquoted and stored at -80°C until further analysis. Each aliquot was frozen and thawed only once. The BM variety was verified and identified by ethnobotanist, Dr. Will McClatchey and voucher specimen was deposited at official herbaria, University of Hawai'i at Manoa, Herbarium (HAW), and labeled as PratibhaBM0001 and PratibhaBM0002 [26].

### Primary Human Adipocyte Cultures

Cryopreserved primary human preadipocytes (catalog # SP-F-SL, lot # SL0028) were obtained from ZenBio (Research Triangle Park, NC) and cultured according to the manufacturer' instructions. The pooled primary preadipocytes were derived from subcutaneous adipose tissues of six female donors with an average age of 43 years and average BMI of 27.25 kg/m^2^. Preadipocytes were maintained in "Preadipocyte Medium" (cat # PM-1, ZenBio) and upon confluence, cells were plated in either 96-well, 24-well or 6-well plates at a density of 13,500, 62,500, or 333,333 cells/well, respectively. Two days after plating, cells were differentiated using "Differentiation Medium" (catalog # DM-2, ZenBio) and considered as Day '0'. Media was changed every two days, 2, 4, 6, 8, 10 and 12. Cells were harvested for analysis on day 14, after which the cells accumulated lipid droplets and were considered as mature adipocytes. In one set of experiments, preadipocytes were treated with BMJ during differentiation from day '0' to day 12 and harvested on day 14 for various analysis. In another set of experiments, when cells were fully differentiated into adipocytes after 14 days, they were treated with 0.5, 1.0 and 2% BMJ for 24 and 48 h.

### Cytotoxicity Measurements

At the end of experimental periods, viability of cells treated with varying BMJ concentrations (0.5, 1.0, 2.0, 5.0 and 10.0%, v/v) was determined using the ATPlite Luminescence Detection Assay System (Cat# 6016941, Perkin Elmer, Boston, MA) according to the manufacturer's protocol. To determine cell cytotoxicity, media was harvested to measure the release of lactate dehydrogenase (LDH) using CytoTox-ONE Homogeneous Membrane Integrity Assay (Cat# G7891, Promega) as described previously [27,28]. Cellular ATP levels are a marker for cell viability since ATP is present in all metabolically active cells and the concentration declines rapidly in apoptotic or necrotic cells.

### Oil Red Staining

Oil Red 'O' staining of adipocytes was performed according to published protocols [29]. In brief, after washing in ice-cold PBS cells were fixed overnight in 10% formalin, washed with water, and stained with 0.3% oil red for 1 h. After thorough washings with water and evaporation of excess water, oil red was extracted in isopropyl alcohol and the absorbance was monitored at 490 nm using Wallac Victor^2 ^1420 Multilabel Counter (PerkinElmer Life Sciences, Boston, MA).

### Cellular Triglyceride (TG) Analysis

Preadipocytes were treated either during differentiation, or after differentiation, with varying concentrations of BMJ as mentioned above. Cells were washed with ice-cold PBS and lysed with 0.5 N sodium hydroxide (NaOH). Cellular TG content was measured using the Infinity TG Liquid Stable Reagent commercial kit (Thermo-DMA, St Louisville, CO, U.S.A.), and the absorbance was read at 540 nm using Wallac Victor^2 ^1420 Multilabel Counter. TG values were normalized to mg of protein as determined by the Bradford method according to the manufacturer's instructions (Bio-Rad Laboratories, Hercules, CA).

### Determination of Lipolysis

Lipolysis was measured as a function of free glycerol released into the medium. In brief, 14 days after differentiation, primary human adipocytes were treated with 0.5, 1 and 2% BMJ for 24 and 48 h. Free glycerol was measured using the commercial glycerol free reagent and glycerol standards according to manufacture's protocol (Sigma, St. Louis, MO).

### Semi-Quantitation of mRNA gene expression

To evaluate the mechanism of reduced adipocyte differentiation and lipid accumulation, mRNA gene expression of peroxisome proliferator-activated receptor gamma (PPARγ), sterol regulatory element-binding protein -1 (SREBP-1) and resistin genes were determined by semi-quantitative reverse transcriptase-PCR (RT-PCR) in preadipocytes treated with varying concentrations of BMJ during differentiation. To further delineate the mechanisms underlying lipolysis, perilipin mRNA expression was determined in adipocytes treated with BMJ for 48 h. Total RNA was extracted using TRIzol reagent (TEL-TEST, Inc., Friendswood, TX). Two μg of RNA was reverse transcribed into complementary DNA (cDNA) and gene expression levels were quantified by two-step RT-PCR using the primer pairs and cycling conditions described in Table 1. Various cycling conditions were initially used to standardize the gene expression profile in the log phase of amplification. Final cycling condition was decided based on half of maximum saturation of the amplicons. The PCR amplicon was size-fractionated on a 2% agarose gel and visualized with ethidium bromide staining. mRNA expressions of various genes were semi- quantified using the Kodak 1D image analysis software that captures the intensity of the amplicons and calculates the pixel counts by normalizing against the background. The intensities of the amplicon were then expressed as a ratio of the gene of interest against a housekeeping gene, GAPDH.

**Table 1:** Oligonucleotide primer sequences used in RT-PCR

Target Gene	Primer sequence	Cycling condition	Size (bp)
PPAR γ2	Forward 5'-GCATGGTGCCTTCGCTGATGC-3' Reverse 5'-AGGCCTGTTGTAGAGCTGGGT-3'	95°C/5 min 95°C/1 min,55°C/1 min 72°C/1 min,72°C/7 min 4°C hold, 33 cycles	340

SREBP-1c	Forward 5'-TTGCGCAAGGCCATCGACTACATT-3' Reverse 5'-ACAAGGGGCTGCTCTGGAAAGGTG-3'	94°C/5 min 94°C/1 min,55°C/1 min 72°C/1 min,72°C/7 min 4°C hold, 26 cycles	238

Perilipin	Forward 5'-TGGAGACTGAGGAGAAGAAG-3' Reverse 5'-ATGTGAGAGGGGAGATGG-3'	94°C/1.5 min 94°C/45 sec,52°C/1.5 min 72°C/1 min,72°C/7 min 4°C hold, 23 cycles	120

Resistin	Forward 5'-TGGTGGTGGTGGCTGTCCTGG-3' Reverse 5'-GAGTGAGATGTGGTCTGGGCG-3'	95°C/2 min 95°C/45 sec,55°C/1.5 min 72°C/45 sec,72°C/7 min 4°C hold, 39 cycles	145

GAPDH	Forward 5'-AGTCAGCCGCATCTTCTTTTG-3' Reverse 5'-CTCCTGGAAGGGAGATGG-3'	Condition adjusted as per the target gene	298

### Statistical Analysis

All statistical analysis was performed using GraphPad Prism, Prism 5 for Windows, version 5.01. Data were expressed as mean values ± SEM. A one-way or two-way ANOVA model was used to compare means between the groups. Each sample was analyzed twice. *Post hoc *pair-wise multiple comparisons were evaluated using the Tukey's Multiple Comparison test or Bonferroni post-test, after ANOVA. Results were considered significant at p < 0.05.

## Results

### BMJ reduces triglyceride (TG) content and increases lipolysis in primary human adipocytes

**Figure 1:**
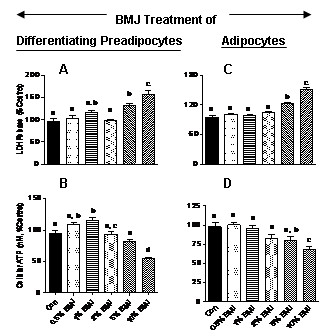
**Cytotoxicity of bitter melon juice in human predaipocytes and adipocytes**. Higher concentrations of BMJ, 5% and 10%, significantly increased LDH release and reduced total ATP levels in both, differentiating preadipocytes and adipocytes. Lower concentrations of BMJ, 0.5% to 2%, did not affect cell viability or cell proliferation in either differentiating preadipocytes or adipocytes. (A) LDH release and (B) cellular ATP levels in preadipocytes treated with BMJ during differentiation. Similarly, LDH release and cellular ATP levels in adipocytes treated with BMJ for 48 h, is depicted in Figures 1D and 1E, respectively. Values represent the mean ± SE (n = 6) of three independent experiments conducted in duplicate. ^a, b, c, d^Mean values with common letters do not differ (p < 0.05).

0.5, 1 and 2% BMJ (v/v) was well tolerated and was not toxic to primary human preadipocytes or adipocytes, while higher concentrations of BMJ (5 and 10%) was cytotoxic as indicated by significantly increased LDH release and reductions in cellular ATP levels. Preadipocytes treated with 5% and 10% BMJ during differentiation demonstrated a significant, 31% and 57%, increase in LDH release when treated, respectively (Figure 1A). Treatment with 5% BMJ was marginally toxic (19% reduction), while 10% BMJ significantly reduced cell viability by 47% (Figure 1B) as measured by cellular ATP levels.

Among adipocytes treated with varying BMJ concentrations for 24 and 48 h, the trends in cellular ATP levels and LDH release were similar to the BMJ-treated differentiating adipocytes. Both, 5% and 10% BMJ significantly increased LDH release by 21% and 51% (Figure 1C). Figure 1D demonstrates that 5% BMJ non-significantly reduced cellular ATP by 20%, while 10% BMJ significantly reduced ATP levels by 32% (p < 0.05) after 48 h. We therefore used 0.5, 1 and 2% BMJ (v/v) in all our subsequent experiments.

**Figure 2:**
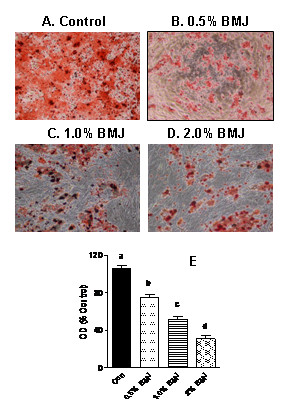
**Effect of bitter melon juice on cellular lipid droplets**. Total cellular lipid contents were significantly reduced in preadipocytes undergoing differentiation when treated with varying concentrations of BMJ (Figures 2A - 2C). Light microscopic pictures of oil red 'O' staining in untreated control adipocytes (A), and preadipocytes treated with - 0.5% BMJ (B), 1% BMJ (C) or 2% BMJ (D), during differentiation (40× magnification). Figure 1E depicts degree of preadipocyte differentiation during treatment as measured by total amount of extracted oil "O" red. Values represent the mean ± SE (n = 6) of three independent experiments conducted in duplicate. ^a, b, c, d^Mean values with common letters do not differ (p < 0.05).

**Figure 3:**
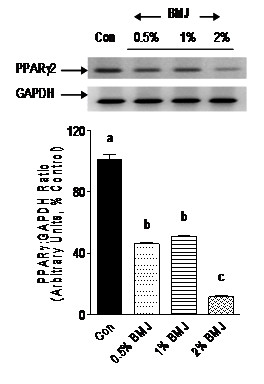
**Effect of bitter melon juice on cellular triglyceride mass**. Cellular triglyceride levels were reduced in (A) differentiating preadipocytes treated with varying concentrations of BMJ during differentiation and (B) adipocytes treated with varying concentrations of BMJ for 24 and 48 h. Figure 3C depicts the increased release of glycerol into the media, when adipocytes were treated with varying concentration of BMJ (0.5% to 2%, v/v) for 24 and 48 h. Values represent the mean ± SE (n = 6) of three independent experiments conducted in duplicate. ^a, b, c, d^Mean values with common letters do not differ (p < 0.05).

Effects of BMJ on cellular differentiation and lipid contents are demonstrated in Figure 2. In contrast to control adipocytes (Figure 2A), cells treated with 0.5% BMJ (Figure 2B), 1% BMJ (Figure 2C) and 2% BMJ (Figure 2D) during differentiation demonstrate a significant reduction in lipid content as observed by reduced oil red staining (Figures 2A to 2D). Figure 2E demonstrates the amount of total lipid content of cells as measured by organic solvent extraction of oil 'O' red from cells. A significant (p < 0.05) dose-dependent reduction in adipocyte differentiation (25 to 70%) was observed in cells treated with BMJ as indicated by reduction in oil 'O' red staining (Figure 2E). Inhibition of differentiation was also accompanied by a significant reduction (p < 0.05) in cellular TG levels by 40 to 70% in cells treated with BMJ during differentiation, as compared to control untreated adipocytes (Figure 3A). Similarly, when adipocytes were treated with BMJ for 24 h and 48 h, cellular TG were significantly reduced (p < 0.05) by 20 to 50% (Figure 3B) with a concomitant increase in lipolysis up to 70% (Figure 3C) (p < 0.05). Treatment of adipocytes with 2% BMJ induced maximum TG reduction and lipolysis after 48 h, Figure 3B and 3C, respectively, but without any significant changes in cellular morphology.

### BMJ reduces mRNA expression of PPARγ, SREBP-1c and resistin

**Figure 4:**
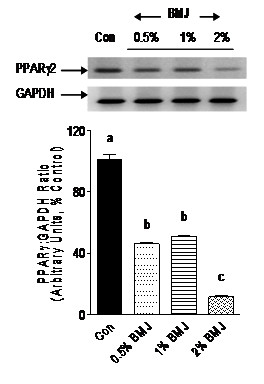
**Effect of bitter melon juice on PPARγ mRNA expression**. Differentiating preadipocytes treated with varying concentrations of BMJ demonstrate a significant reduction in PPARγ mRNA gene expression. Bar graphs depict the densitometry scans of PPARγ amplicons (348-bp) and are expressed as a ratio to GAPDH amplicon (298-bp) intensity. Data are expressed as a percentage of the control (set as 100%) and the values represent the mean ± SE (n = 6) of three independent experiments analyzed in duplicate. ^a, b, c^Mean values with common letters do not differ (p < 0.05).

**Figure 5:**
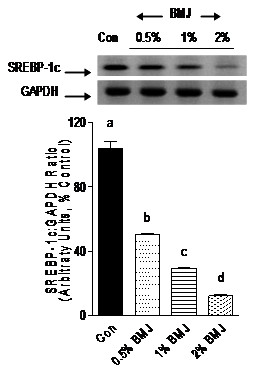
**Effect of bitter melon juice on SREBP-1c mRNA expression**. Differentiating preadipocytes treated with varying concentrations of BMJ (0.5% to 2%, v/v) demonstrate a significant reduction in SREBP-1c mRNA gene expression. Bar graphs depict the densitometry scans of SREBP gene amplicons (238-bp) and are expressed as a ratio to GAPDH amplicon (298-bp) intensity. Data are expressed as a percentage of the control (set as 100%) and the values represent the mean ± SE (n = 6) of three independent experiments analyzed in duplicate. ^a, b, c^Mean values with common letters do not differ (p < 0.05).

**Figure 6:**
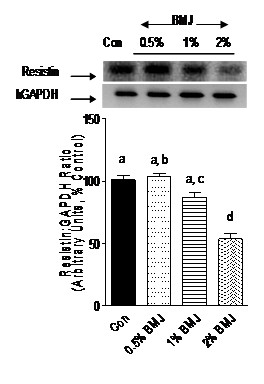
**Effect of bitter melon juice on resistin mRNA expression**. Differentiating preadipocytes treated with varying concentrations of BMJ (0.5% to 2%, v/v) demonstrate a significant reduction in resistin mRNA gene expression. Bar graphs depict the densitometry scans of resistin gene amplicons (145-bp) and are expressed as a ratio to GAPDH amplicons (298-bp) intensity. Data are expressed as a percentage of the control (set as 100%) and the values represent the mean ± SE (n = 6) of three independent experiments analyzed in duplicate. ^a, b, c^Mean values with common letters do not differ (p < 0.05).

Adipocyte transcription factors such as PPARγ and SREBP-1c, as well as adipocytokine, resistin, play a crucial role in adipocyte differentiation, adipogenesis, and accumulation of cellular lipid droplets. The fact that BMJ reduced adipocyte differentiation prompted us to investigate the effects of BMJ on mRNA expression of PPARγ, SREBP-1c and resistin. Preadipocytes treated with 0.5, 1 or 2% BMJ during differentiation demonstrated a significant reduction (p < 0.005) in PPARγ mRNA expression, 50 to 80%, as compared to control untreated adipocytes (Figure 4). Similarly, a significant (p < 0.05) dose-dependent reduction in SREBP-1c mRNA expression was observed, further confirming an anti-adipogenic effect of BMJ (Figure 5). However, reduction in mRNA expression of resistin gene in preadipocytes treated with 0.5 and 1.0% BMJ during differentiation was not significant as compared to untreated control cells. In contrast, treatment with 2% BMJ demonstrated a significant reduction of 45% (p < 0.05) in resistin gene expression (Figure 6). Overall, maximum inhibition of adipocyte differentiation and transcription factors was observed with 2% BMJ.

### BMJ inhibits perilipin mRNA expression

**Figure 7:**
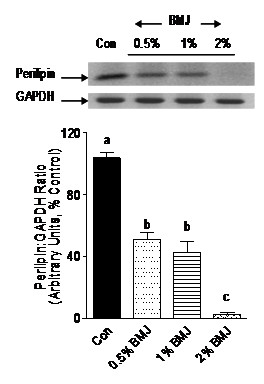
**Effect of bitter melon juice on perilipin mRNA expression**. Adipocytes treated with varying concentrations of BMJ (0.5% to 2%, v/v) demonstrate a significant reduction in perilipin mRNA gene expression. Bar graphs depict the densitometry scans of 120-bp perilipin gene amplicons and are expressed as a ratio to GAPDH amplicon (298-bp) intensity. Data are expressed as a percentage of the control (set as 100%) and the values represent the mean ± SE (n = 6) of three independent experiments analyzed in duplicate. ^a, b, c^Mean values with common letters do not differ (p < 0.05).

Formation of lipid droplets in adipocytes as well as lipolysis is regulated by a membrane protein surrounding the lipid droplets, perilipin, which is also a downstream target of PPARγ in adipocytes. BMJ-associated reduction in cellular TG (Figure 3B) and increased lipolysis (Figure 3C) in adipocytes, was accompanied by a significant reduction (p < 0.05) of perilipin gene mRNA expression in adipocytes treated with BMJ for up to 48 h (Figure 7). There was no significant change in morphological appearance of the cells or cell death, but reduction in lipid size droplets.

## Discussion

Bitter melon is used to treat diabetes and its complications in traditional Chinese and Ayurvedic medicine. We and others have demonstrated that besides improving glucose and lipid metabolism [26,30-32], BMJ is also effective in improving hyperlipidemia in diabetic and obese rodents as well as reduces body weights in mice fed high-fat diet [24,25,33]. However, the effects of bitter melon on human adipocytes have not been investigated. Transition of undifferentiated fibroblastic preadipocytes into mature adipocytes involves differential regulation of adipogenic genes as well as lipid accumulation. Increase in fat mass is a result of not only increase in adipocyte number due to proliferation, but also induction of differentiation that stimulates mitotic clonal expansion and irreversible commitment to differentiation [34]. Therefore, reduction in fat mass during weight loss may involve inhibition of adipogenic process and lipid accumulation due to dedifferentiation, lipid mobilization (lipolysis) and/or programmed cell death (apoptosis). Higher concentrations of BMJ at 5% and 10% demonstrate significant reduction in cell viability as indicated by reduced cellular ATP levels and increased LDH release. It is therefore possible that higher concentration of BMJ induces apoptosis in maturing preadipocytes and adipocytes, which requires further investigation.

During normal adipogenesis, growth arrest after confluency is followed by mitotic expansion and adipogenic signals to induce differentiation and lipid accumulation [35]. Mitotic clonal growth is accomplished by increased expression of CCAAT/enhancer binding protein beta (C/EBPβ) which further stimulates the expression of two transcription factors, C/EBPα and PPARγ involved in adipogenesis and lipogenesis [36]. Our data indicates that lower concentrations of BMJ (0.5% to 2%) has no effect on growth arrest or cell death, but may possibly promote dedifferentiation of maturing preadipocytes via reduction in PPARγ mRNA gene expression and reduction in lipogensis. Although we did not measure C/EBPα, it is possible that effects of BMJ are mediated upstream via C/EBPβ. To our knowledge, this is a first study to elucidate the effects of BMJ on primary human adipocyte differentiation and analyze the adipogenic gene expressions. PPARγ, a nuclear hormone receptor, plays a critical role in peripheral glucose homeostasis and energy metabolism and has been implicated in modulating adipogenesis and insulin sensitivity *in vivo*. Anti-diabetic agents such as rosiglitazone are PPARγ agonist and improve glucose metabolism by increasing adipogenesis thereby leading to secondary weight gain [37]. Recent studies indicate that partial PPARγ antagonism by various plant extracts may be beneficial in improving insulin sensitivity and may also inhibit adipocyte differentiation and lipid accumulation [34,38,39]. In the present studies, BMJ significantly inhibited adipogenesis in differentiating preadipocytes with a concomitant reduction in PPARγ gene expression.

We further observed that SREBP-1c, another regulator of lipid homeostasis and adipogenesis [40] was significantly reduced by BMJ in primary human adipocytes with a simultaneous reduction in cellular lipid content indicated by reduced oil 'O' red staining. Studies by Huang et al. indicated that BM powder, prepared from whole fruit, inhibited adipocyte hypertrophy in diet-induced obese (DIO) rats possibly due to reduction in the expression of lipogenic genes including fatty acid synthase (FAS), acetyl CoA carboxylase (ACC 1), lipoprotein lipase (LPL) and adipocyte fatty acid-binding protein (aP2) in epididymal fat, which are down-stream targets of SREBP-1c [25]. However, Huang et al. did not observe any effects on either PPARγ or SREBP-1c mRNA expression in the adipose tissue of rats fed HFD and BM, but suggested possible effects of BM on either the protein levels or post-transcriptional modifications of these genes [25].

Adipocytokines such as leptin and adiponectin, involved in food intake, energy metabolism and weight gain have been demonstrated to be regulated by BMJ in cell culture and rodent studies [41,42]. It has been recently demonstrated that adipocytokine, resistin, is involved in human adipocyte differentiation [12]. In our study, BMJ significantly reduced mRNA expression of resistin in primary human adipocytes treated during differentiation. Similarly, Shih et al. [42] also demonstrated a significant reduction in adipose resistin expression in mice fed HFD with BMJ. Among obese individuals, the increased plasma resistin has been demonstrated to positively correlate with changes in BMI and visceral fat as well as mRNA expression in abdominal fat [43,44]. The fact that plasma resistin levels are reduced in subjects with moderate weight loss [45] and our data indicating reduced resistin expression in primary human adipocytes, supports the hypothesis that BMJ possess an anti-adipogeneic potential in humans.

Additional mechanisms for reduced adiposity in BMJ-fed rodents may include increased levels of plasma catecholamines that are known to promote lipolysis in adipose tissue [24]. Perilipins are the most abundant lipid droplet-specific proteins that influence adipocyte TG storage and accumulation by regulating lipolysis [46]. They are synthesized on free ribosomes rather than on endoplasmic reticulum-bound ribosomes and require post-translational assembly onto lipid droplets [46]. Perilipins also regulate lipolysis by modulating the access of hormone sensitive lipases (HSL) to the surface of lipid droplet [47]. Although post-transcriptional phosphorylation of perilipin via G-protein coupled receptor-activated protein kinase A (PKA) is associated with increased lipolysis in human adipocytes [48], studies with TNFα indicate that adipocyte lipolysis was also associated with inhibition of perilipin mRNA expression [49]. Similarly, our data also indicate that lipolytic activity of BMJ is possibly associated with significant inhibition of perilipin mRNA expression. Since perilipin is a down-stream target of PPARγ, it is highly possible that BMJ may interfere with ligand-binding domain of PPARγ or may inhibit endogenous PPARγ ligands and block PPARγ target genes. Alternatively, we cannot overrule the possibility that BMJ can directly phosphorylate perilipin proteins by activation of G-protein coupled receptors.

The range of BMJ concentrations used in the current study is comparable to those used in earlier studies [26]. However, the exact effective and safe dose of BMJ for human consumption is unknown and requires further clinical evaluation. BMJ contains many active chemicals including charantin (a steroid glycoside), polypeptide "p" or plant insulin (a 166 residue insulin mimetic peptide) [50], glycosides such as mormordin, vitamin C, carotenoids, flavanoids and polyphenols [51,52]. Our unpublished observations and those by Islam et. al. [53] indicate that BMJ contains various polyphenols including quercetin and gallic acids. Quercetin has been demonstrated to inhibit adipogenesis and induce apoptosis in 3T3-L1 mouse adipocytes [54,55], while gallic acid is known to increase the number of early and late apoptotic 3T3-L1 cells in culture via loss of mitochondrial membrane potential [56]. We therefore speculate that insulin-mimetic peptides as well as polyphenols such as quercetin and gallic acid may in part be responsible for anti-adipogenic properties of BMJ. Recent studies indicate that cucurbutanoid compounds are the active principals of BMJ which possess hypoglycemic properties [57], but the exact active ingredients possessing hypolipidemic properties are unknown.

Overall our studies demonstrate that BMJ inhibits primary human adipocyte differentiation by reducing PPARγ, SREBP and perilipin mRNA gene expression and by increasing lipolysis. In animal studies, although BMJ reduced adiposity, Shih et al. demonstrated that adipose PPARγ mRNA expression was significantly increased in DIO mice fed BMJ extracts and HFD, as compared to both, HFD-fed and control mice [42], while Huang et al. did not observe any effects on adipose lipolysis in rats fed HFD with BM [25]. These differences are possibly due to differences in the model system (primary human adipocytes vs. rodents), BM preparations and/or experimental conditions.

A few clinical studies have investigated the anti-diabetic effects of BMJ in humans [58-62], however, long-term studies testing the effects of BMJ on glucose and lipid metabolism, body weight as well as identifying the pharmacokinetics and effective dose of BMJ is warranted, before it can be recommended as an effective alternative and/or complementary therapy. Regardless of its therapeutic effects, use of BMJ with other hypoglycemic or hypolipidemic agents must be performed with caution and under medical supervision due to its hypoglycemic properties [63,64] and excessive consumption must be avoided due to possible side effects such as diarrhea [61].

## Conclusions

Bitter melon was effective in reducing lipid accumulation in primary human adipocytes by regulating adipogenic transcription factors and adipocytokine gene expression. Future studies are warranted to test the effects of BM consumption on weight loss and body fat accumulation in humans.

## Abbreviations

ACC 1: acetyl CoA carboxylase; aP2: adipocyte fatty acid-binding protein; BM: bitter melon; BMI: body mass index; BMJ: bitter melon juice; C/EBP: CCAAT/enhancer binding proteins; DIO: diet-induced obesity; FAS: fatty acid synthase; *Insig-1*: insulin-induced gene 1; LPL: lipoprotein lipase; NEFA: non-esterified fatty acids; PPARγ: peroxisome proliferator-associated receptor γ; RT-PCR: reverse transcriptase-PCR; SREBP-1c: sterol regulatory element-binding protein 1c; T2D: type 2 diabetes; TAG: triacylglycerol; TG: triglycerides.

## Competing interests

The authors declare that they have no competing interests.

## Authors' contributions

PVN conceived and designed the study as well as analyzed and interpreted the data, and wrote the manuscript. Y-KL performed RT-PCR and enzymatic assays for data acquisition and VRN was involved in data analysis and critically revising the manuscript for important intellectual content. PVN has primary responsibility for final content. Authors have read and approved the final manuscript.

## Pre-publication history

The pre-publication history for this paper can be accessed here:

http://www.biomedcentral.com/1472-6882/10/34/prepub
